# Astragalus improves intestinal barrier function and immunity by acting on intestinal microbiota to treat T2DM: a research review

**DOI:** 10.3389/fimmu.2023.1243834

**Published:** 2023-08-10

**Authors:** Min Su, Ting Tang, Weiwei Tang, Yu Long, Lin Wang, Meiling Liu

**Affiliations:** ^1^ Hunan Key Laboratory of the Research and Development of Novel Pharmaceutical Preparation, Changsha Medical University, Changsha, China; ^2^ Department of Biochemistry and Molecular Biology, School of Basic Medicine, Changsha Medical University, Changsha, China

**Keywords:** Astragali Radix, active ingredients, type 2 diabetes mellitus, intestinal microbiota, immunity, metabolism

## Abstract

Diabetes is a significant chronic endocrine/metabolism disorder that can result in a number of life-threatening consequences. According to research, the gut microbiota is strongly linked to the development of diabetes, making it a viable target for diabetes treatment. The intestinal microbiota affects intestinal barrier function, organism immunity, and thus glucose metabolism and lipid metabolism. According to research, a disruption in the intestinal microbiota causes a decrease in short-chain fatty acids (SCFAs), alters the metabolism of bile acids (BAs), branched-chain amino acids (BCAAs), lipopolysaccharide (LPS), and endotoxin secretion, resulting in insulin resistance, chronic inflammation, and the progression to type 2 diabetes mellitus (T2DM). Astragali Radix is a medicinal herb of the same genus as food that has been extensively researched for treating diabetes mellitus with promising results in recent years. Polysaccharides, saponins, flavonoids, and other components are important. Among them, Astragaloside has a role in protecting the cellular integrity of the pancreas and liver, can leading to alleviation of insulin resistance and reducing blood glucose and triglyceride (TC) levels; The primary impact of Astragalus polysaccharides (APS) on diabetes is a decrease in insulin resistance, encouragement of islet cell proliferation, and suppression of islet β cell death; Astragali Radix flavonoids are known to enhance immunity, anti-inflammatory, regulate glucose metabolism and control the progression of diabetes. This study summarizes recent studies on Astragali Radix and its group formulations in the treatment of type 2 diabetes mellitus by modulating the intestinal microbiota.

## 1 Introduction

Diabetes mellitus is a set of endocrine illnesses defined by hyperglycemia, which can damage tissues and organs in the body due to a variety of factors (1). Type 2 diabetes mellitus (T2DM) is frequent in the middle-aged and elderly, and it accounts for more than 90% of all diabetes mellitus types (2). T2DM is strongly linked to the intestinal microbiota, according to research. The main aspects are mainly in the immune system, energy metabolism, intestinal screen function, intestinal barrier function (3). Numerous studies have shown that the occurrence of T2DM is closely related to the intestinal microbiota, which is increasingly recognized as a key factor in the development of T2DM. Diabetic patients have moderate gut ecological dysbiosis, with significant changes in the composition and diversity of their intestinal microbiota. In T2DM patients, the relative abundance of opportunistic pathogenic bacteria *Clostridium* increased whereas the relative abundance of butyrate-producing bacteria *Roseburia*, *Faecalibacterium*, and *Eubacterium* decreased (4). Dysbiosis of the intestinal microbiota may result in the release of endotoxins and the promotion of a chronic inflammatory response in the body, which may lead to insulin resistance and, eventually, diabetes (5). In terms of the immune system, intestinal microbiota and its metabolic derivatives, such as lipopolysaccharide (LPS) and short chain fatty acids (SCFAs), not only act directly on intestinal epithelial cells to modulate the immune system and so maintain immune homeostasis, but also activate downstream pathways via free fatty acid receptors and Toll-like receptors (TLRs), which are found throughout the body. TLRs and free fatty acid receptors are found throughout the body and have the ability to activate downstream pathways and have regulatory effects on the pancreas, insulin target organs, and other tissues (6, 7). Therefore, regulating intestinal microbiota can improve glucose metabolism levels, insulin resistance and increase insulin sensitivity, which is important for T2DM treatment.

It has been found that using Chinese herbal medicine to address intestinal microbiota abnormalities in the body is useful in the prevention and treatment of T2DM (8). Astragali Radix is a Chinese plant originated in Northern China that first appears in the Shennong Ben Cao Jing (9) and serves as one of the most commonly used single herbs in hypoglycemic herbal medicine. In traditional Chinese medicine (TCM), Astragali Radix can be paired with other herbs, such as ginseng and atractylodes, to participate in the composition of a variety of Chinese medicine compound. In China, Astragali Radix is not only commonly used in TCM, but is also known to be a tonic with beneficial effects on qi (maintains basic life activities and involved in the composition of the body) and yang (which fuels the body’s all physiological functions and metabolism). These functions are considered to be the original application of the herb in immunomodulation (10). And the important role of Astragali Radix in modulating immune function in humans and experimental animals is inextricably linked to the intestinal microflora and intestinal barrier function (3, 7). A variety of chemical substances have been isolated and identified from Astragali Radix, including saponins, flavonoids and polysaccharides. Astragali Radix has been used for thousands of years to treat diabetes, and its “thirst quenching” effect and disease treatment have been mentioned in several Chinese medical treatises. Modern medical research has shown that Astragali Radix can regulate the intestinal microbiota of T2DM patients, optimize the structure of intestinal microbiota, regulate the immune system and intestinal barrier function, and these are the possible mechanisms for its effective treatment of T2DM (11). Using intestinal microbiota as the target, we review the research on the interaction between Astragali Radix and intestinal microbiota for the treatment of T2DM, with the aim of providing ideas for the study of the mechanism between T2DM and intestinal microbiota and clinical development of herbal compound preparations and new drugs for T2DM treatment, as well as providing more safe and effective options for the T2DM treatment.

## 2 The relationship between intestinal microbiota and T2DM

**Figure 1:**
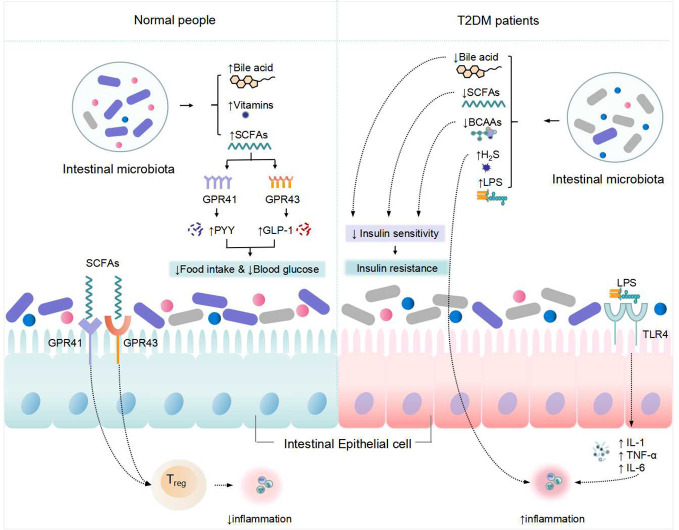
Changes in metabolism of the intestinal microbiota and its associated with T2DM. "↑" indicates an increase, "↓" Indicates a decrease.

The intestinal microbiota, can be considered as one of the human organs, is critical to human health. It has even been described as the “eighth organ” of humans. Intestinal microbiotas’ positive effects on the host include energy metabolism, gut protection, immune regulation, and brain function (12). Under normal conditions, the host and intestinal microbiota are in a mutually beneficial symbiotic relationship, maintaining a benign dynamic balance. When the host’s internal environment changes or pathogenic microorganisms invade, this balance will be disrupted, causing intestinal microbial, which can lead to a series of obesity and other metabolic diseases, inflammation, diabetes, etc (13). Changes in intestinal microbiota are linked to the progression of T2DM, and studies have shown that transplantation of fecal bacteria into germ-free mice in T2DM mice caused diabetic-like changes (14), and conversely, transplantation of fecal bacteria from normal mice In contrast, transplantation improves metabolic status in db/db mice (15). Because of alterations in the number of diverse bacteria in the intestinal microecology, the structure and distribution of intestinal microbiota in T2DM patients varies dramatically from those in normal persons. Allin et al. (16) showed that the abundance of *Akkermansia muciniphila*, *Clostridium* in decreased abundance and *Streptococcus, Sutterella*, *Ruminococcus*, *Dorea*, in increased abundance, with *Ruminococcaceae*, *Lachnospiraceae* showing a particularly strong negative correlation with fasting glucose and insulin levels. Everard et al. (17) showed that Bacteria which promote inflammation such as *Bacteroides* were more common in the feces T2DM patients. Larsen et al. (18) found that in diabetic patients The relative abundance of *Clostridia*, *Firmicutes* was significantly reduced and *Betaproteobacteria* was highly enriched. The ratios of *Bacteroides-Prevotella/C.coccoides-E*., *Bacteroidetes/Firmicutes* and plasma glucose concentration showed a significant positive correlation with plasma glucose concentration. Zhou et al. (19) found that the intestinal microbiota of rats changed dynamically with the progression of T2DM, with the abundance of *Akkermansia muciniphila, Lactobacillu* and butyric acid-producing-related bacteria *Turicibacter* decreasing as T2DM progressed, while the abundance of *Allobaculum*, *Ruminococcus* increased in abundance (as shown in **Table 1**; **Figure 1**). Some studies have also proposed that changes in the makeup and diversity of the intestinal microbiota may influence the severity of T2DM.

**Table 1:** Comparison of the intestinal microbiota and its function in patients with T2DM patients and healthy controls.

	Healthy controls	T2DM patients
**intestinal microbiota**	SCFA-producing bacteria *clostridiales* *F.prausnitzii* *R.inulinivorans* *R.intestinalis*	*C.symbiosum* *C.ramosum* *C.hathewayi* *E.lenta*
**Functions**	Bacteria chemtaxis Butyrate biosynthesis Metabolism of Cofactors and vitamins	Membrane transport of sugars Resistance to oxidative stress Biosynthesis of hydrogen sulfide Degradation and metabolism of xenobiotics BCAAs and LPS synthesis

### 2.1 The relationship between intestinal microbiota and SCFAs production

In the intestine, intestinal microbiota digest oligosaccharides, polysaccharides, peptides, proteins, and glycoproteins to create SCFAs, primarily butyric acid, propionic acid, and acetic acid. SCFAs have been proven in studies to regulate blood glucose and affect intestinal function (20). SCFAs regulate host metabolic health directly through a variety of mechanisms related to energy expenditure, appetite regulation, immune regulation, and glucose homeostasis, and its important functions are mainly in the maintenance of colonic epithelial energy supply, inhibition of pathogenic bacteria growth, regulation of the immune system and glucose and lipid metabolism (21). *Bacillus*, *Clostridium* and *Bifidobacterium* are the most frequent bacteria that produce SCFAs. *Anaplasma* is capable of producing acetate. Propionate is generated by a few dominating genera, including the basal species of mucinophilic *Ackermannia*, and it is also produced via the succinate route by the common and polymorphic *Anaplasma* Butyrate is generated by an anaerobic *Clostridium* species group. Butyrate, for example, has been shown to increase mitochondrial activity, prevent metabolic endotoxemia, inhibit the secretion of pro-inflammatory factors such as interleukin-6 (IL-6) and tumor necrosis factor α (TNF-α), improve insulin sensitivity, has anti-inflammatory potential, and improves intestinal barrier function (22). Acetate can also be converted to cholesterol or fatty acids, and propionate can be involved in gluconeogenesis (23). By boosting protein kinase signaling in the liver and muscle, SCFAs can improve insulin sensitivity. Gprotein-coupledreceptor-41 (GPR-41) and GPR-43 are both found in human islets, where activation of GPR43 promotes insulin secretion and activation of GPR41 acts as an inhibitor, both of which work together to maintain insulin release order and glucose homeostasis (24). SCFAs can also stimulate insulin secretion by interacting with G protein-coupled receptors that enhance Glucagon-likepeptide-1 (GLP-1) and Peptide-YY (PYY) (25). SCFAs are not only metabolites of anti-inflammatory bacteria, but they also work as immune boosters, assisting the host in fighting inflammation. Increased levels of microbial gut-derived SCFAs are regarded to be favorable to health, while gut microbial imbalance caused by intestinal inflammation and decreased SCFAs may be connected to diabetes etiology (26). intestinal microbiota disruptions may alter the synthesis and metabolism of SCFAs *in vivo*, resulting in decreased insulin sensitivity and disrupted insulin secretion, leading to the development of T2DM.

### 2.2 The correlation between the level of BCAAs and intestinal microbiota

Branched chain amino acids (BCAAs) are the basic amino acids that make up proteins, including leucine, isoleucine, and valine, and their levels in the blood alter insulin sensitivity. Horiuchi et al. (27) discovered that decreasing the protein content of mice’s food decreased insulin secretion, but increasing BCAAs in the diet restored insulin secretion and brought it back to normal levels. However, high doses of BCAAs may lead to insulin resistance. High levels of BCAAs can act as an upstream trophic signal by initiating the mTOR signal transduction pathway and inhibiting the expression of the translocator Kruppel-like factor 15 (KLF15), a key regulator of lipid, glucose, amino acid, and bile acid metabolism, resulting in increased insulin sensitivity and glycogen synthesis (28, 29). The synthesis of BCAAs in the body is intimately tied to intestinal microbiota, and the levels of BCAAs and their metabolites in the body also affect the flora (30, 31). As a result, gut flora can influence insulin sensitivity by influencing BCAAs levels, which play a vital part in the development of T2DM.

### 2.3 The correlation between LPS secretion and intestinal microbiota

LPS, a major component of the cell wall of Gram-negative bacteria, causes an inflammatory response (32, 33). TLRs are natural immune system receptors that play a key role in triggering an inflammatory response. TLR4 and NF-κB play critical roles in the LPS-mediated signal transduction pathway (34). TLR4 receptors detect and bind LPS, which activates the NF-κB signal transduction pathway, causing the production of proinflammatory molecules such as IL-6 and TNF-α, causing inflammation and increasing insulin resistance. Intestinal microbiota alteration can result in the production of high amounts of LPS and inflammatory chemicals, leading in chronic and persistent inflammation, structural damage, malfunction, and even apoptosis of pancreatic-cells, Consequently, insufficient insulin secretion and insulin resistance occur, leading to the development of T2DM (35).

### 2.4 The correlation between metabolism of BAs and intestinal microbiota

Bile acids (BAs) are an essential component of bile and the main result of cholesterol metabolic conversion in the liver. Bile acids have a vital role in intestinal absorption, transportation, and distribution of fat-soluble vitamins and lipids. It controls the network of metabolic signal transduction pathways involved in in the metabolism of glucose, lipid, drug, and energy, and facilitating lipid and fat-soluble vitamin absorption (36). Bile acid control necessitates the cooperation of the liver, the intestine, and gut bacteria. Cholesterol is processed by the liver to form primary BAs and by the action of intestinal microbiota to form secondary BAs. BAs function primarily as signaling molecules and ligands in glucose metabolism via the farne-soid X receptor (FXR) signal transduction pathway. Bile acids can activate G protein-coupled bile acid receptor 5 (TGR5) and FXR while also inhibiting gluconeogenic gene expression (37). FXR has been demonstrated to change the makeup of intestinal bacteria, boost bile acid production, and improve glucose tolerance in obese T2DM mice when the vertical trocar stomach is removed (37). TGR5 activation in intestinal L cells causes GLP-1 release, which increases insulin secretion, decreases glucagon secretion, improves liver and pancreatic function, and reduces insulin resistance and metabolic inflammation caused by a high fat diet (38). It is worth noting that the majority of the research on the aforementioned pathways has been done in rodent models. In contrast, there are differences in the composition and hydrophobicity of the bile acid metabolic pool between humans and rodents, which have a large impact on bile acid signaling properties.

### 2.5 Intestinal microbiota’s function in organism immunity

While boosting metabolic activities in the body, intestinal microbiota also influences host physiological functions such as immune system and intestinal epithelial cell formation, pathogen defense, and tissue homeostasis maintenance. The mucosal barrier of the intestine is a complex system combining tissue, cellular, chemical, and immunological levels. The endocrine system of the intestine and the mucosa of the intestine have a “one loss, one gain” connection. The intestinal tract is an essential component of the human immune system, intestinal inflammation is closely related to the intestinal microflora and, some researches has linked immunological problems to insulin resistance and other pathological characteristics of T2DM (39, 40). Damage to the intestinal mucosa can allow germs and their toxins to enter the body’s important tissues and organs via the bloodstream, triggering a metabolic inflammatory response. T2DM is frequently accompanied with injury to the intestinal mucosa, and the inflammatory response may also play a role in the development of T2DM (40). Intestinal immune cells are mostly found in the connective tissue of the lamina propria of the gut mucosa, which serves as a medium for communication between intestinal bacteria and the host, and are constantly impacted by intestinal microecology. The intestinal barrier normally prevents the stimulation of the immune system by pathogenic microorganisms; however, in the case of intestinal epithelial cell breakdown, abnormal tight junctions, and increased intestinal permeability, enterobacteria, viruses, and their metabolites can penetrate deeply into the lamina propria via the epithelium, stimulating adaptive immune responses and causing the release of various inflammatory factors (41). Wu et al. (42) demonstrated the most substantial effect of LPS on intestinal mucosal immunity. Endotoxemia caused changes in the ratio of the number of various lymphocytes in rat intestinal tissues. The intestinal immune system and the intestinal mucosa have a very strong connection, indicating an interdependent relationship.

The large amount of inflammatory factors released by immune disorders activates inflammatory signaling pathways in the intestinal mucosa, resulting in abnormal cell function or apoptosis and necrosis, which increases LPS and inflammatory factor leakage into the bloodstream and triggers a systemic chronic inflammatory response (43). Furthermore, intestinal immunological inflammation can affect the release of intestinal endocrine hormones such as TNF-α, which can suppress GLP-1 secretion via a receptor-dependent route, causing irregularities in glucose metabolism in the body (43).

**Figure 2:**
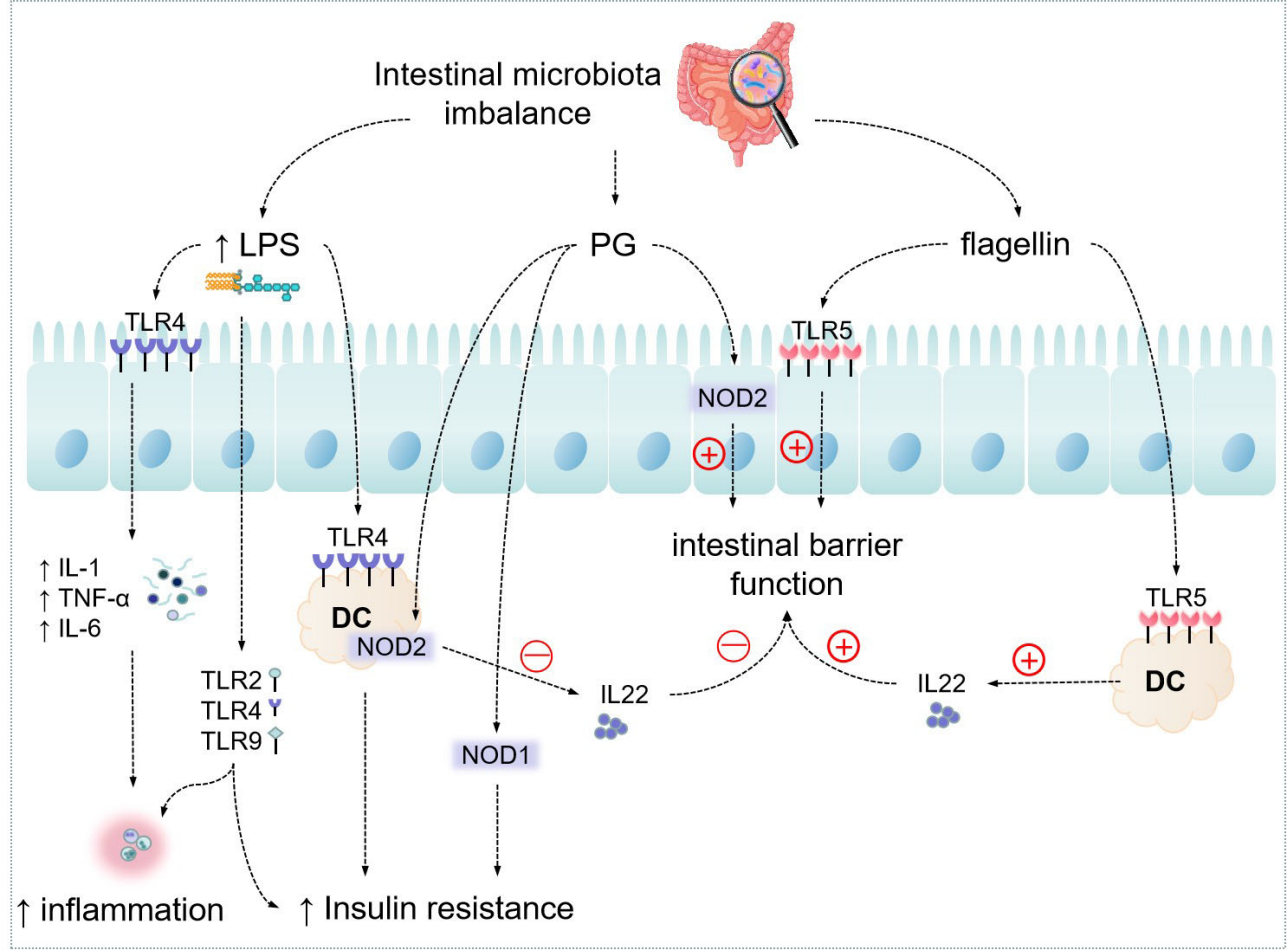
Effect of Intestinal microbiota on host metabolism through messenger molecules. "↑" indicates an increase.

The key cell types involved in immunological control in the immune system include Gut-associated lymphoid tissue (GALT), T-lymphocytes, B-lymphocytes, Group 3 intrinsic lymphoid cells, macrophages, and dendritic cells (41). Pattern recognition receptors (PRRs) are a class of receptors associated with the immune recognition of viruses, bacteria, fungi, or parasites and are part of the host’s innate defense system (44). LPS and multiple PAMPs activate PRRs, which sense microbes and infection factors and signal defense responses. Four classes of PRRs including transmembrane proteins, such as C-type lectin receptors (CLRs) and TLRs; cytoplasmic proteins, such as NOD-like receptors (NODs) and retinoic acid inducible gene-like receptors (RLRs) have been found (45). TLRs are the most well researched PRRs, and their recognition activates genetic programs via post-transcriptional control of transcription factors and mRNAs (46). Different microbial components of pathogens can mediate different responses of TLRs. TLRs are present in immune cells and somatic cells such as intestinal epithelial cells (47). TLR2 detects bacterial lipoproteins, whereas TLR4 recognizes bacterial LPS (as shown in **Figure 2**) (48). Their activation causes antigen-presenting cells to become activated, therefore combining with the autoimmune response and triggering signaling cascades in anticipation of protect against microbial invaders or heal injured tissues. Although this inflammatory reaction is necessary for eradication of infection, overactivity of TLRs can disturb immunological homeostasis, and persistent production of chemokines and pro-inflammatory cytokines may increase the likelihood of inflammation and immune disease (49, 50). Chassaing et al. (51) found that TLR5 is activated by bacterial flagellin, and mice lacking TLR5 develop colitis or metabolic syndrome, which is due to changes in the intestinal microbiota. This means that altered microflora leading to inflammatory responses can interfere with the expression of PRRs.

Furthermore, multiple studies have revealed that commensal bacteria’s metabolic byproducts, such as SCFAs, influence the immunological response of gut-associated lymphoid tissues (GALTs) via epigenetic processes, operating through self-defense and the immune evasion by commensal bacteria (41).

## 3 Potential mechanism of Astragali Radix in the treatment of T2DM

Normal intestinal microbiota function in the body maintains the metabolic balance between microbes and the body. If this equilibrium is upset, the alteration and disorder of intestinal microbiota will result in an abnormal body metabolism in the body, including the formation of diabetes. Probiotics are living bacteria that benefit the host’s health. Prebiotics can be selectively absorbed and transformed into helpful health compounds by the host flora. Several studies have found that probiotics, prebiotics, and other microbial alteration therapies have an influence on appetite and body weight (3). The addition of probiotics to replenish reduced taxa in T2DM therapy may enhance gut metabolic activities (e.g., immunomodulation, SCFAs synthesis, and bile acid metabolism regulation) (52). *Lactobacillus rhamnosus*, *Lactobacillus griseus*, *Lactobacillus casei*, *Lactobacillus lactis*, *Streptococcus thermophilus*, *Lactobacillus bulgaricus*, and *Bifidobacterium lactis* have been found in studies to have anti-diabetic efficacy (53). In patients with metabolic diseases, fecal transplantation (FT) improves the sensitivity to insulin, enhances gut microbial diversity, and dramatically boosts butyric acid-producing bacteria (54, 55). Clinical trials have shown that FT can increase the sensitivity to insulin in normal person and effectively delay the decline of β-cell function and halt the progression of diabetes in diabetic patients, but the benefits are temporary and highly dependent on the individualized response of the host (56, 57). It can be demonstrated that proper intestinal microbiota modulation may regulate glucose metabolism, reduce insulin resistance, improve and stabilize blood glucose levels, and successfully cure diabetes. Astragali Radix membranaceus, a Chinese herbal medication, another aspect, has a positive impact in terms of controlling the intestinal microbiota in the body and can successfully cure T2DM in a variety of ways, including decreasing insulin resistance and lowering blood glucose.

### 3.1 Mechanisms of Astragali Radix in the treatment of T2DM acting on the intestinal microbiota

Diabetes mellitus is classified as Xiaokezheng in TCM, and its etiology is mostly owing to a lack of yin and fluids, as well as a predominance of dryness and heat. Diabetes mellitus is intimately linked to intestinal microbiota abnormalities in the body, according to Chinese medicine; however, intestinal microbiota disorders and spleen malfunctions are mutually causative. According to certain research, the status of normal intestinal microbiota is directly associated to the spleen (58). The ancient people believed that the spleen and stomach were the foundations of human survival, and that the spleen and stomach modified the body’s qi, blood, and vitality through eating. so the normal operation of the spleen and stomach is critical in the balance of intestinal microbiota. *Bifidobacteria*, *Lactobacilli*, and other beneficial bacteria in the intestinal tract, which can decompose food residues, metabolize them in the body in various ways, and finally transform them into essential substances to maintain body nutrition, allowing the human intestinal tract to remain balanced and healthy. Thus, managing intestinal microbiota can enhance spleen and stomach function, repair and ameliorate intestinal mucosal damage, restore intestinal wall permeability, regulate and translocate harmful bacteria in the intestinal tract, and enhancement of autoimmunity, all of which are important in combating of T2DM (59).

In recent years, many traditional Chinese herbs have been tried in combating of T2DM (60), and Astragali Radix can treat thirst primarily by “inducing clear qi to reach the lungs, harmonizing with the inhaled qi and transforming it into water, encouraging the upward movement of fluid in the stomach, and regulating the qi of the lower jiao.” Astragali Radix is thought to be a natural hypoglycemic agent with broad application potential is critical in combating of diabetes (61). Astragali Radix is a somewhat warm, sweet-tasting herb that belongs to the spleen and lung meridians and has a variety of actions include helping the body, tonifying qi and elevating yang, stimulating water retention, lowering swelling, and discharging pus and supporting sores (61). Polysaccharides, saponins, flavonoids, and other multi-class active components in Astragali Radix have strong preventative and therapeutic benefits on T2DM, including reducing insulin resistance, enhancing insulin sensitivity, antioxidant, and anti-inflammatory properties (61). By controlling intestinal microbiota abnormalities, increasing the immunological barrier function of intestinal mucosa, improving the body’s immunity, and regulating metabolites, Astragali Radix can exhibit anti-diabetic actions (61). In terms of pharmacological mechanism, Astragali Radix can increase tyrosine kinase activity, improve insulin signal transmission, and aid in combating of diabetes and its complications (61). Wang et al. (61) Astragali Radix adjusts the ratio of SCFAs-producing bacteria such as *Flavonifractor*_and *Alloprevotella* and inflammation-associated bacteria such as *Alistipes* and *Deferribacteres*. Li et al. (11) used 16S rRNA sequence determination to examine the effects of Astragali Radix on the regulation of intestinal microbiota in type 2 diabetic mice and discovered that Astragali Radix significantly improved the diversity and abundance of intestinal microbiota from diabetic mice, inducing an improving in the abundance of *Bifidobacterium* and *Lactobacillus*, thereby controlling body weight and blood glucose in type 2 diabetes mice.

### 3.2 Astragalus formulas in the treatment of T2DM acting on the intestinal microbiota

Astragali Radix as the main herb, together with various other herbal formulas, has also shown remarkable results in diabetes control through acting on the intestinal microbiota (as shown in **Table 2**). Cao et al. (62) evaluated the safety and efficacy of the new formula Qi-Jian combination (QJ) (Astragali Radix, Yang Mei, Ge Gen, and Huang Lian) in combating of type 2 diabetes by metabolomics, and showed that it can regulating blood glucose and triglyceride (TC) levels, reducing liver and kidney damage, and increasing production of SCFAs and decreasing production of BCAAs. PCoA analysis of intestinal microbiota indicated that QJ therapy significantly increased the number of Akkermansia and Bacteroidetes, reduced the number of Firmicutes (62). The system pharmacology paradigm also demonstrated that QJ worked via the PPARA, AKT1, and TP53 proteins (62). It was determined that QJ significantly reduced T2DM, and this impact was associated with changed metabolite profiles and intestinal microbiota. Xie et al. (63) showed by 16S rRNA sequence determination that the various ameliorative effects of Pi-Dan-Jian-Qing decoction (PDJQ) (Pseudostellaria heterophylla, Astragali Radix, Rhizoma atractylodes, Radix scrophulariae, Puerariae lobatae radix, Scutellaria, Coptis chinensis, Salvia miltiorrhiza, dried ginger) on T2DM, including improving hyperglycemia, hyperlipidemia and insulin resistance, improving liver and kidney function and regulating inflammatory response and oxidative stress. The mechanism of PDJQ for T2DM may be related to the improvement of intestinal microflora dysbiosis, regulation of tryptophan and histamine metabolism and citric acid cycle. The mechanisms of on T2DM are likely linked to an improvement in the dysbiosis of intestinal microbiota and modulation of tryptophan metabolism, histamine metabolism, and the TCA cycle. In addition, ELISA results showed that PDJQ decreased the levels of TNF-α, IL-1β and IL-6. PDJQ can improve the clinical symptoms of spleen deficiency and dampness-heat caused by T2DM, and can reduce both postprandial blood glucose (PBG) and fasting blood glucose (FBG) (64). The Jin-Xiao-Xiao-Ke decoction (JXXKD), first reported in Wai Tai Mi Yao Fang which was writing by Wang Tao during the Tang Dynasty (AD 752), is a traditional prescription used to treat symptoms “Xiao Ke,” which is known as T2DM in modern medicine. This prescription was used to cure “Xiao Ke” and considerably reduce symptoms like polyuria and thirst. Animal studies revealed that JXXKD dramatically reduced polyuria symptoms, lowered FBG protein levels, and improved liver fat deposition in diabetic rats (65). Guo et al. (66) mentioned in their study about the mechanism of treatment of T2DM that Drug Pair of Astragali Radix and Dioscoreae Rhizoma modulates the metabolism of BCAAs (e.g., leucine and isoleucine) and SCFAs (e.g., butyric acid and propionic acid) *in vivo*. low-density lipoprotein (LDL), total cholesterol (TC) and Lipid levels were reduced and high-density lipoprotein (HDL) levels were increased in T2DM rats after these treatments. In addition to this, they found that Drug Pair of Astragali Radix and Dioscoreae Rhizoma can regulate the disturbance of energy metabolism and reducing insulin resistance.

**Table 2:** Therapeutic effect of Astragalus group formula on T2DM.

	Changes in intestinal microflora or metabolite levels		Therapeutic effect	Document
	Promotes	Reduces		
**Qi-Jian combination**	*Bacteroidetes* *Akkermansia* SCFAs	*Firmicutes* FBG, TC BCAAs	liver and kidney damage ↓ inflammation ↓ alleviates type 2 diabetic symptoms	29391233
**Pi-Dan-Jian-Qing decoction**	*Lactobacillus* *Blautia* *Bacteroides* *Desulfovibrio* *Akkermansia*	FBG, PBD TNF-α, IL-1β, IL-6 *Prevotella*	inflammatory ↓ Oxidative ↓ insulin resistance ↓ alleviates type 2 diabetic symptoms	34938667
**Jin-Xiao-Xiao-Ke decoction**	*−*	FBG, TC urine	Oxidative ↓ alleviates type 2 diabetic symptoms	36353458
**the Drug Pair of Astragali Radix and Dioscoreae Rhizoma**	SCFAs HDL	BCAAs LDL, TC	insulin resistance ↓ Oxidative ↓ regulate the disturbance of energy metabolism alleviates type 2 diabetic symptoms	31717456

"↓" indicates a decrease or weakening.

## 4 Mechanism of the active ingredients of Astragali Radix membranaceus in the treatment of T2DM by regulating the intestinal microbiota

Astragali Radix contains a variety of active ingredients, among which saponins and polysaccharide flavonoids have been reported most frequently, and all these types of ingredients have some therapeutic effects on T2DM, and their specific molecular mechanisms are as follows (as shown in **Table 3**).

**Table 3:** Molecular mechanisms of the active ingredients of Astragali Radix membranaceus in the treatment of T2DM.

Active ingredients of Astragali Radix	Changes in metabolite levels	Related signaling pathways	Therapeutic effect	Document
**Saponins**	FBG ↓ TC ↓ SCFAs ↑	PI3K/AKT AMPK/SIRT1 NADPH oxidase/ROS/Akt/NF-κB	oxidative ↓ insulin resistance ↓ diabetes-induced nephropathy ↓ alleviates type 2 diabetic symptoms	21693217 32628809 19610026 21555978 23719694 29207011 30245468 24718766
**Polysaccharides**	SCFAs ↑	Endoplasmic reticulum stress-related signaling pathways glucose transporters and sweet taste receptors/GLP-1/GLP-1 receptor signaling pathways	intestinal microbiota disorders ↓ intestinal inflammation ↓ alleviates type 2 diabetic symptoms	29258283 36386641 36368354 36106494 32832560 19524131 29257218 17950099
**Flavonoids**	GOT, GPT ↓ TG,TC ↓ LDL ↓ HDL ↑	PGC1/AMPK	oxidative ↓ inflammation ↓ promoting insulin secretion restoring the intestinal barrier effectively treat diabetic kidney damage alleviates type 2 diabetic symptoms	30830898 20735814 35151348 33953787

"↑" indicates an increase, "↓" indicates a decrease or weakening.

### 4.1 Saponins

Astragaloside (AS), one of the main active ingredients of Astragali Radix, which chemical nature is a kind of cycloartane-type triterpene glycoside chemical. Recent studies have shown that AS has hypoglycemic effects and may alleviate diabetic complications through several potential molecular mechanisms. Gong et al. (67) reported that AS significantly lowered blood lipid, glucose, insulin resistance, and oxidative stress levels, In T2DM rats. According to histology results, AS has a role in protecting the cellular integrity of the pancreas and liver. AS modulates the type and number of intestinal microbiota and increases butyric acid levels in diabetic mice (67). AS treatment of diabetes was found to be achieved by modulating two signaling pathways, PI3K/AKT and AMPK/SIRT1, and confirmed using insulin-resistant HepG2 cells and inhibitors (67). Cheng et al. (68) discovered that AS-IV can participate in the elevation of glucocorticoid expression in rats. Lv et al. (69) reported that the amount of glucose-6-phosphatases and glycogen phosphatases in the liver was lowered in mice with T2DM treated with AS-IV, leading to alleviation of insulin resistance and decreases in blood glucose and TC levels. He & Gong et al. (67, 70) also reported that Astragaloside IV may promoting butyric acid production by modulating the intestinal microbiota profile. Zhang et al. (71) discovered that AS-IV administration might ameliorate metabolic syndrome induced by high fructose diet in rats, resulting in lower blood pressure and improved insulin resistance. Qi et al. (72) showed that AS-IV can alleviate the Glycated albumin causes epithelial-mesenchymal transition (EMT) in the cell of Normal Rat Kidney-52E line via redox balance modulation. Similarly, He et al. (73) found that AS-IV protected rat kidney from hyperinsulinemia. The mechanisms were inhibition of TNF-α and IL-1 overproduction, reduction of oxidative stress, enhancement of transient receptor potential channel (TRPC6) expression, and inhibition of extracellular-signal-regulated kinase 1/2 (ERK1/2) activation. AS-IV reduces Diabetes-induced nephropathy by ameliorating oxidative stress and suppressing the expression of inflammatory genes driven by NF-κB in human mesangial cells (74, 75).

### 4.2 Polysaccharides

Like Saponins, Astragalus polysaccharides (APS) are one of the main active components of Astragali Radix. It is very closely related to intestinal microbiota. APS has a positive regulatory effect on intestinal microbiota, reduces insulin resistance and improves islet secretory cells in T2DM. It has been shown that after gavage of APS in db/db diabetic mice, the specific value of the *phylum Bacteroides* and *Thick-walled Bacteroides* in the organism was increased significantly, and the abundance of *Lactobacillus* and *Sartorius* increased, while *Spirochaete mucocephalus* and *Treponema* were growth inhibited, confirming that APS can delay glucose diffusion, treat diabetes and repair its tissue damage (76). APS not only has a positive regulatory effect on intestinal microbiota disorders, but also regulates body metabolism, improves the intestinal milieu, and plays a vital role in a wide range of properties as an example anti-inflammatory, immunomodulatory and anti-diabetic properties (77). Ming et al. (78) found that APS significantly increased serum concentrations of SCFAs, including butyric and propionic acids, by increasing the abundance of SCFA-producing genera such as *Akkermansia*, *Coprococcus*, and *Oscillospira* in the intestine. APS may control immune function by boosting cytokine release, promoting immune cell proliferation, and influencing immunoglobulin (Ig) production and immunological signaling (79). APS decreased oxidative stress and inflammation levels, alleviated Glucose and lipid metabolism abnormalities, and avoided organ destruction in T2DM mice, according to Chen et al. (80), the APS might preserve the intestinal barrier by reducing oxidative stress and intestinal inflammation stress, as well as inhibiting the pathogenic bacteria *Shigella* and promoting the development of good bacteria *Allobaculum* and *Lactobacillus*. Antibiotic test have shown that the possible glucose-lowering mechanism of APS is the restoration of the intestinal barrier by altering particular intestinal bacteria.

The pathophysiology of T2DM is mostly related to insulin resistance, islet β cell dysfunction, and insulin insufficiency. Tanase et al. (81) found that normal islet β cell actions include decreasing insulin resistance, regulating lipid metabolism, boosting insulin production, and lowering blood glucose levels. Wang et al. (82) showed through their study that APS may lower blood glucose and enhance insulin sensitivity by lowering endoplasmic reticulum stress in T2DM patients. Its method may entail decreasing ATF6 activation (caused by endoplasmic reticulum stress), reversing ATF6 translocation in cells, and inhibiting the high expression of a certain protein phosphatase. The above changes reduce the stress on the liver endoplasmic reticulum, which enhances insulin sensitivity (82). Furthermore, Wei et al. (83) found that APS can decreasing T2DM insulin resistance by maintaining or increasing miRNA-203a-3p expression, lowering the glucose-regulated protein (GRP78) expression, and modulating the endoplasmic reticulum stress-related signaling pathway protein expression. According to Seino et al. (84), APS can promotes anaerobic oxidation in muscle tissue and attenuates insulin resistance. APS has also been found in several studies to stimulate cell differentiation and induce the secretion of adiponectin, increases T2DM rat glucose transporter 4 (GLUT4) expression in adipocytes and AMPK expression in liver tissue, downregulations resistin protein and mRNA in adipocytes (85–87). Another report said that APS can improve the condition of type 2 diabetic rats by modulating the intestine-pancreatic axis and related signaling pathways (88).

Collectively, the primary impact of APS on diabetes is a decrease in insulin resistance, encouragement of islet cell proliferation, and suppression of islet β cell death. Its pharmacological impact is primarily mediated through altering the expression of associated genes and proteins (86). The growth environment of intestinal microorganisms has a high correlation with the function of the spleen and stomach. The above studies suggest that APS has various effects on anti-inflammation, promotion of ulcer tissue proliferation and repair of damaged blood vessels from the perspective of the spleen. APS plays a crucial role in the treatment and improvement of diabetes and associated disorders by regulating the microecological environment *in vivo*.

### 4.3 Flavonoids

Astragali Radix membranaceus contains flavonoids, which have been shown in recent studies to have immune-boosting, anti-inflammatory, glucose metabolism-regulating, and diabetes progression-controlling properties. According to Zhang et al. (89), a flavonoid component of Astragali Radix may effectively treat diabetic kidney damage in db/db mice by inhibiting IB and NF-B p65 and decreasing inflammatory markers in blood. Furthermore, after treatment of this component, blood glucose levels dropped in db/db obese mice, which is assumed to be connected to the anti-inflammatory impact. In mice treated with this component, serum TC levels were lowered, and glucose intolerance and insulin resistance were regulated (90). After oral administration of flavones from Astragali Radix chemistry, enriching the intestinal microbiota in STZ/high-fat diet-induced diabetic mice, flavones from Astragali Radix were shown to lower fasting blood glucose and food intake (90). *In vitro* experiments revealed that Astragali Radix flavonoids boosted HT22 cell survival and preserved the Normalization of the intestinal barrier function in the CaCO_2_ monocyte layer, a mechanism involving the PGC1/AMPK pathway (91). Flavones from Astragali Radix significantly reduced cholesterol and cholesteryl esters, TC, glutamic-oxaloacetic transaminase (GOT), glutamic-pyruvic transaminase (GPT) and LDL levels while elevating HDL levels in both *in vivo* and *in vitro* experiments. Cholesterol 7α-hydroxylase (CYP7A1) and glucose transporter 2 (GLUT2) expressions were increased significantly in the treatment of flavones from Astragali Radix, and TGR5 and FXR played key roles in regulating lipid and bile acid metabolism (92).

## 5 Summary and prospect

In China, TCM has been widely used in the clinical treatment of “Xiaoke” for a long time. The reason why TCM acts directly on the intestinal microbiota is inextricably linked to its oral administration. The intestinal microbiota transforms and promotes the absorption of TCM components, while at the same time, TCM can alter intestinal microbiota abundance and numbers and influence its metabolites (93). This article reviews the relationship between intestinal microorganisms and the occurrence and progression of T2DM, as well as how Astragali Radix and Astragalus formulas can be utilized to effectively treat T2DM. Astragali Radix, as a medicinal and food herb, can positively regulate the intestinal microorganisms, promotes probiotic colonization, inhibit the growth of pathogenic microorganisms, influence the differentiation and apoptosis of intestinal cells, thereby improve the intestinal barrier function and immune function.

As found by Liu et al. (76), APS altered the intestinal microbiota, such as increasing the number of *Bacillus thuringiensis* and *Lactobacillus lactis*, which improved cognitive performance of diabetic mice. By influencing the proliferation of intestinal bacteria, Astragali Radix can affect metabolites and their activities such as SCAAs, BCAAs, LPS, BAs, and others. These metabolite concentrations, which are strongly connected to intestinal barrier function, blood glucose and lipid levels, insulin sensitivity, and inflammatory levels, may account for the role of Astragali Radix in a range of metabolic diseases, including diabetes (11). The polysaccharides, flavonoids and saponins in Astragali Radix, all of which act on the intestinal microbiota, are now recognized as a possible mechanism for the treatment of diabetes mellitus. Changes in the populations of many pathogenic microorganisms are a major trigger for diabetes mellitus (62). Intestinal probiotics can regulate the metabolism of herbal ingredients and other substances in the body by acting on intestinal mucin to maintain normal intestinal barrier function. It may alleviate T2DM by regulating microbial metabolism, alleviating insulin resistance, maintaining intestinal barrier function, and reducing oxidative stress, among other mechanisms. Previous studies can help us to recognize the history and current research status of Astragali Radix in the treatment of diabetes, and lay the foundation for the future clinical application of Chinese medicine in the treatment of diabetes.

However, the intestinal microbiota is a dynamically changing and complex population and herbal medicines may bring adverse effects (94, 95). Therefore, in-depth research is needed in the study of herbal medicines acting on the gut flora to modulate the immune system for the treatment of metabolic diseases. Especially considering the individual variability, cum the effect of differences in the composition of gut microbiota and immunity in different people (96). Our research on similar signaling processes in different bacteria should continue to expand with a view to establishing standardized therapies using relevant signaling pathways or targeting molecules and better applying herbal therapeutic techniques in clinical practice.

## Author contributions

MS and ML conceived the paper. MS and ML analyzed the relevance of the literature and wrote the article. TT, WT, YL, and LW revised the figures and reviewed the article. All authors contributed to the article and approved the submitted version.
